# Study on the Influence of the Surface Altered Layer on Fracture Initiation and Load-Bearing Capacity of Gouged Pipelines

**DOI:** 10.3390/ma19030462

**Published:** 2026-01-23

**Authors:** Hui Yang, Can He, Enming Zhang, Fuxiang Wang, Yuguang Cao, Ying Zhen

**Affiliations:** 1 PipeChina Institute of Science and Technology, Tianjin 300457, China; 2 College of Mechanical and Electronic Engineering, Shandong University of Science and Technology, Qingdao 266590, China; hec0107@163.com (C.H.);; 3 College of Pipeline and Civil Engineering, China University of Petroleum, Qingdao 266580, China

**Keywords:** gouge-induced altered layer, X70 pipeline steel, gouged pipeline, GTN damage model, fracture initiation, load-bearing capacity

## Abstract

To clarify the influence of gouge-induced altered layers on fracture initiation and load-bearing capacity of pipelines, X70 pipeline steel is taken as the research object. The geometry and partition of the altered layer are first determined by means of a micro-Vickers hardness array and a threshold criterion, and its mechanical parameters are then obtained from small-scale tensile tests. The altered layer is subsequently embedded into a finite element model of a gouged pipe as an independent material domain, and the Gurson–Tvergaard–Needleman (GTN) damage model is employed to simulate damage evolution and crack propagation under pure internal pressure and combined internal pressure and tensile loading. The results indicate that, compared with the base metal, the yield strength and ultimate tensile strength of the altered layer increase by about 39% and 47%, respectively, while the elongation to failure decreases from 16% to 1.8%, exhibiting a typical “high-strength–low-ductility” behavior. When the altered layer is considered, the fracture initiation location under pure internal pressure shifts from the base metal to the altered layer, and the burst pressure decreases from 19 MPa to 16.5 MPa. Under the combined internal pressure and tensile loading, the peak load changes little, whereas the ultimate displacement is reduced by about 26.5%, leading to a marked loss of pipeline ductility. These findings demonstrate that the gouge-induced altered layer has a significant effect on the fracture initiation pressure, failure mode, and load-bearing characteristics of gouged pipes. Modeling it as an independent material domain in finite element analysis can more realistically capture the failure behavior and safety margin of gouged pipelines, thereby providing a more reliable theoretical basis for improving integrity assessment criteria for externally damaged pipelines.

## 1. Introduction

In recent years, as long-distance oil and gas transmission pipelines have moved toward higher pressure, higher steel grades, and more complex service environments, the integrity assessment of pipelines containing mechanical damage has attracted sustained attention. Mechanical damage such as gouges not only alters the local geometry of the pipeline, but also changes the near-surface material properties through severe plastic deformation, resulting in an altered layer with a hardness gradient and degraded ductility [1,2]. Full-scale burst tests have also shown that the critical failure pressure of combined defects containing gouges is significantly lower than that of defects with similar geometry but without gouges [3,4], indicating the dominant role of the altered layer in structural failure behavior.

In engineering practice, surface gouges are commonly caused by third-party mechanical interference (e.g., excavation equipment, construction tools, or impact/dragging of hard objects during installation and maintenance). Such events typically generate a localized U/V-shaped notch with a rounded tip, where severe plastic deformation occurs and a hardened–embrittled altered layer forms. Therefore, in this study, we reproduced a representative gouge using a controlled gouging device and adopted the measured gouge profile (depth/width) obtained by 3D scanning to ensure the defect geometry is realistic and repeatable.

Recent studies on mechanically damaged pipelines have made substantial progress in both experimental characterization and assessment methodology. In addition to extensive burst-test databases and empirical/semi-empirical correlations for dent–gouge failure, several modified failure assessment diagrams and assessment procedures have been proposed to improve the prediction accuracy for combined defects and to reduce the conservatism of existing codes. In parallel, with the increasing availability of high-fidelity experimental data and numerical simulations, data-driven approaches such as Gaussian-process-regression-based models have been proposed to improve burst-capacity prediction for pipelines containing dent–gouges [5].

At the material level, fracture initiation of modern high-strength pipeline steels under multi-axial stress states has also been extensively investigated. A growing number of recent studies have emphasized the necessity of incorporating stress triaxiality and Lode parameter effects into ductile fracture models, and have developed or calibrated uncoupled fracture criteria and micromechanics-based models (e.g., GTN-type models) for X70/X80 pipeline steels to improve the prediction of crack initiation and subsequent crack growth [6,7,8,9]. These advances provide a robust theoretical basis for linking microvoid damage evolution with macroscopic crack initiation.

Despite these advances, the gouge-induced altered layer can be regarded as a locally “hardened but embrittled” material zone, and its mechanical response and fracture initiation behavior can be distinctly different from that of the base metal. Specifically, the altered layer is typically characterized by a pronounced hardness gradient, and its yield strength and tensile strength are markedly higher than those of the base metal, whereas its uniform plasticity and fracture ductility are substantially reduced. Therefore, treating gouged pipelines as homogeneous structures and applying only geometric parameters or empirical strength reduction factors may lead to non-conservative predictions, especially for high-grade pipelines and under multi-axial loading conditions.

Nevertheless, quantitatively assessing the influence of the altered layer on fracture initiation and load-carrying capacity of gouged pipelines in engineering practice is challenging, primarily because the altered layer itself is difficult to test and characterize due to its small thickness and the steep property gradients. Consequently, many existing numerical studies still assume uniform material properties and introduce an empirical reduction factor for the gouged region, which lacks a clear physical basis. Moreover, systematic modeling of fracture initiation in a coupled “altered-layer–base-metal” system is lacking, and even fewer studies have quantitatively linked this behavior to the crack initiation pressure and load-carrying degradation of gouged pipelines.

In summary, several key scientific questions remain insufficiently addressed: (i) how to experimentally obtain the mechanical response of the altered layer and the base metal beneath the gouge, and to determine the geometric scale and hardness gradient of the altered layer; (ii) how to establish a fracture initiation model that can capture the damage evolution and crack initiation of the altered layer and the base metal under multi-axial stress states; and (iii) how to quantify the impact of the altered layer on the fracture initiation pressure and the load-carrying capacity of gouged pipelines under internal pressure and combined internal pressure plus tensile loading conditions.

## 2. Specimen Preparation and Experimental Procedures

### 2.1. Preparation of Gouged Material

The test material is a pipeline steel conforming to the API 5L X70 specification. This steel grade exhibits excellent strength–toughness matching and has been widely used in major national projects such as the West–East Gas Pipeline and the China–Russia natural gas pipeline [10]. The pipe has an outer diameter of 1422 mm and a wall thickness of 23.2 mm. Its chemical composition (mass fraction) is as follows: C 0.068%, Si 0.18%, Mn 1.68%, S 0.0004%, P 0.0093%, Cr 0.25%, Ni 0.011%, Mo < 0.002%, V 0.0036%, Nb < 0.045%, Ti < 0.013%, with the balance Fe. Plate specimens with dimensions of 300 mm × 150 mm were cut from the pipe body.

**Figure 1:**
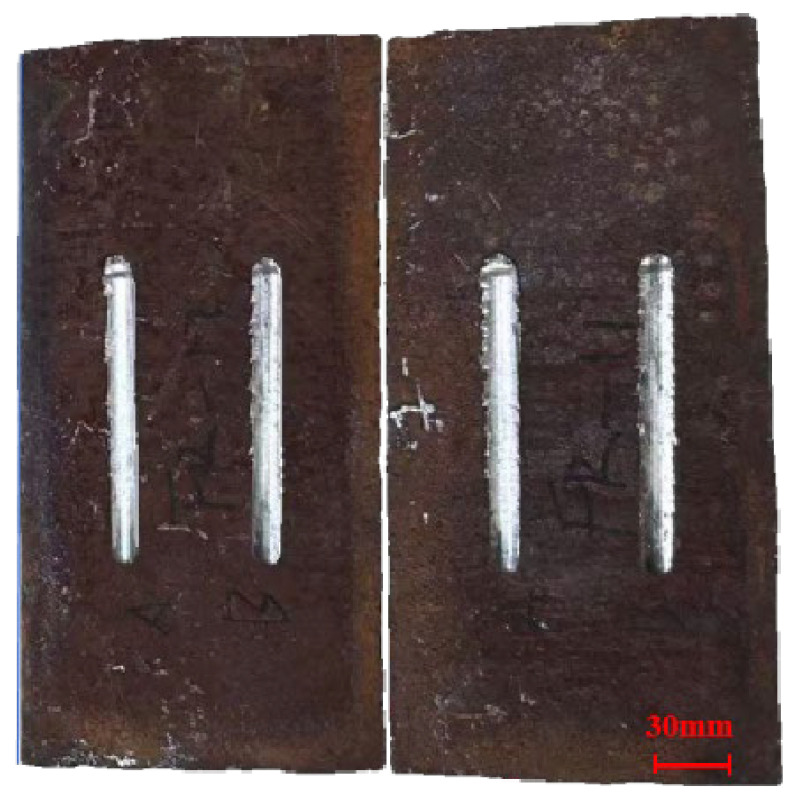
Pipeline with a scratch defect.

To obtain repeatable and representative gouge defects, surface gouges were machined using a purpose-built (custom-made) gouging device. The gouging tool has a tip angle of 120° and a tip curvature radius of 16 mm. During machining, a load-controlled mode was adopted: a vertical force of 90 kN was applied, and the tool was moved along the axial direction of the pipe at a constant speed of 10 mm/s to form the gouge. The resulting gouged specimen is shown in Figure 1. Measurements indicate that the gouge length is 120 mm, the width is 10 mm, and the depth is 0.82 mm. Two parallel gouges were machined on each plate specimen mainly for sampling efficiency and repeatability: one gouge was used for microhardness mapping and tensile coupon extraction, and the other served as a replicate. The spacing between the two gouges was selected to be sufficiently larger than the gouge width so that their plastic/damage zones do not overlap; thus, each gouge can be treated as an independent defect.

### 2.2. Microhardness Tests

**Figure 2:**
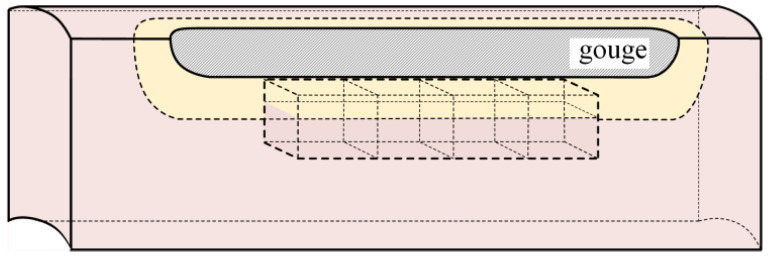
Location for hardness sampling.

In accordance with the requirements for hardness gradient testing, four block specimens with dimensions of 10 mm × 10 mm × 10 mm were consecutively cut from the longitudinal section through the center of the gouge bottom. Microhardness measurements were carried out using a TH783 digital Vickers hardness tester (Beijing TIME High Technology Ltd., Beijing, China) with a test load of 10 N, following the standard GB/T 4340.1-2009 [11]. First, three measurement paths were selected on both the upper and lower surfaces of each specimen, with a path spacing of 1 mm and a spacing of 0.5 mm between adjacent indents, in order to obtain the hardness distributions of the base metal and the altered layer. Subsequently, hardness tests were performed on three characteristic cross-sections of the specimens, with a spacing of 1 mm between measurement points in the horizontal direction and 0.3 mm between points along the depth direction. The specific sampling locations of the microhardness specimens are shown in Figure 2.

**Figure 3:**
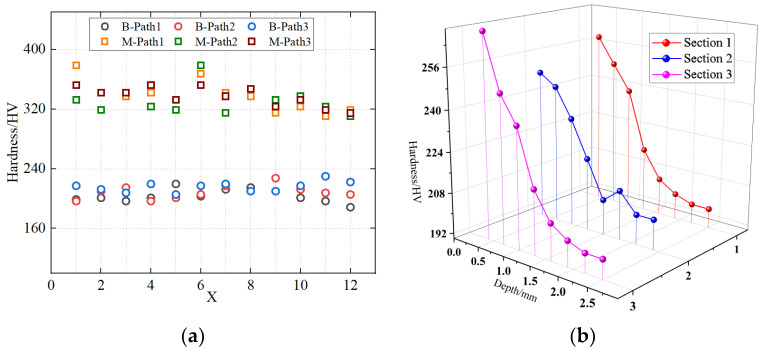
Hardness test results. (**a**) Hardness profile characteristics. (**b**) Variation of hardness with depth in the cross-sectional direction.

The hardness distribution characteristics on the upper surface (altered-layer side) and lower surface (base metal side) of the hardness specimens are shown in Figure 3a, where the altered layer and base material are denoted by M (metamorphic layer) and B (base material), respectively. The hardness test results indicate that the hardness values in the base metal region range from 188 to 230 HV, whereas the hardness of the altered layer is significantly higher, all above 300 HV, with a maximum value of 378 HV.

The relatively large scatter in the base metal microhardness is mainly attributed to local microstructural heterogeneity of X70 steel (e.g., ferrite–bainite banding/constituent distribution at the indentation scale) and point-to-point variability inherent to Vickers microhardness testing (surface preparation, exact indentation positioning, and local constraint effects). To reduce the influence of scatter on layer identification, we use the upper bound of the base metal range (230 HV) as a conservative threshold when delineating the altered layer. The horizontal hardness measurements on the three characteristic cross-sections of the hardness specimens were averaged to obtain the hardness variation along the depth direction of each section, as shown in Figure 3b. It can be seen that, for all three sections, the hardness decreases from the altered-layer side toward the base metal side, eventually dropping to the base metal hardness level and becoming stable. The hardness decreases with depth because gouging introduces a strong plastic-strain gradient. Severe near-surface deformation causes pronounced work hardening, whereas the strain rapidly decays toward the interior; thus, the microstructure and hardness progressively recover to the base metal level and become stable. This continuous hardness gradient can be used to determine the interface position between the altered layer and the matrix region [12,13]. For X70 pipeline steel, in order to reduce the influence of scatter in the base metal hardness, 230 HV was selected as the critical hardness value for distinguishing the altered layer from the base metal. When the hardness at a test point is greater than 230 HV, the corresponding region is considered to have undergone a significant change in properties and thus belongs to the altered layer; when the hardness is lower than this threshold, the material is assumed to essentially retain its original properties and is classified as the matrix region. Based on statistical averaging of the measurement data from the three sections, the thickness of the gouge-induced altered layer in this X70 pipeline steel was determined to be 1.08 mm.

### 2.3. Tensile Tests

**Figure 4:**
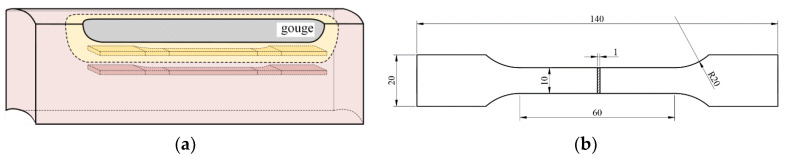
Sampling locations and dimensions of the tensile specimens. (**a**) Schematic of the sampling location. (**b**) Geometric configuration of the tensile specimen.

Based on the microhardness test, the thickness of the altered layer was determined to be 1.08 mm. Accordingly, thin-plate tensile specimens with a thickness of 1 mm were cut from the gouge bottom along the gouge centerline, and additional specimens were cut from the remote base metal region away from the gouge to obtain the mechanical properties of the altered layer and the base metal for comparison and modeling. The geometry of the tensile specimens is shown in Figure 4b. Given the property gradient across the altered-layer thickness, the measured stress–strain response of the 1 mm coupon is interpreted as an effective (homogenized) constitutive behavior averaged over the altered-layer thickness.

For altered-layer specimens, 1 mm thick sheets were sliced parallel to the gouge-bottom surface along the gouge centerline. The sheet top surface coincided with the gouge bottom, and the 1 mm thickness captured the near-surface region beneath it; so, the tensile gauge section mainly sampled the altered layer (~1.08 mm thick). To reduce edge effects, the gauge section was aligned with the centerline and placed sufficiently far from the gouge shoulders. For base metal specimens, 1 mm thick sheets were taken from a remote region far from the gouge where microhardness had stabilized at the base metal level, ensuring no influence from gouge-induced plastic deformation.

Using the digital image correlation (DIC) technique, image acquisition was carried out. Prior to testing, a speckle pattern was sprayed onto the surface of the tensile specimens, and strain was measured at an interval of 1 s per frame. The tests were carried out on a Shimadzu universal testing machine with a maximum load capacity of 300 kN, under displacement control at 1 mm/min, in accordance with GB/T 228.1–2021 “Metallic materials-Tensile testing-Part 1: Method of test at room temperature” [14]. The yield strength *R*_p0.2_, ultimate tensile strength *R*_m_ and elongation after fracture *A* were obtained from the engineering stress–strain curves.

**Figure 5:**
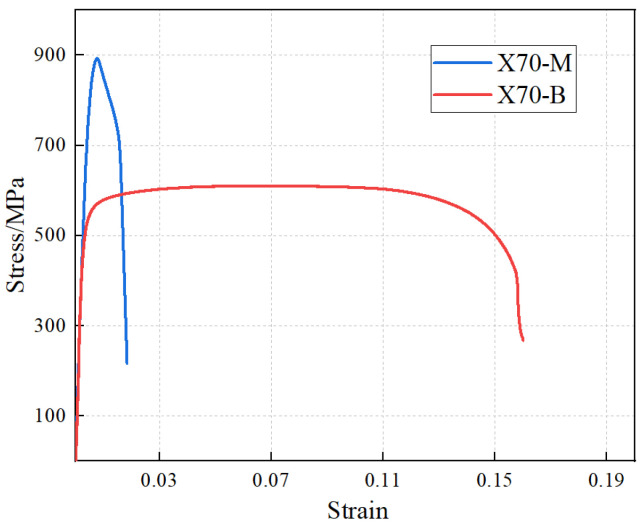
Stress–strain curve from the tensile test.

The stress–strain curves of the tensile specimens from the altered layer are shown in Figure 5. The tensile test results indicate that, compared with the base metal, the yield strength and ultimate tensile strength of the altered layer at the gouge bottom are significantly increased: the yield strength rises from 541 MPa to 752 MPa, and the ultimate tensile strength from 610 MPa to 899 MPa. In contrast, the elongation after fracture of the altered layer is greatly reduced, decreasing from 16% to 1.8%, while the elastic modulus remains nearly unchanged, as expected. The tensile properties of the material are summarized in Table 1.

The markedly different tensile responses of X70-M and X70-B originate from the gouge-induced altered layer. Severe plastic deformation during gouging causes significant work hardening, which elevates the yield and ultimate strengths. Meanwhile, the altered layer contains a high density of defect, which accelerates damage accumulation and suppresses uniform plastic deformation, leading to the observed “high-strength–low-ductility” behavior.

## 3. Finite Element Model Development

### 3.1. Determination of Damage Parameters

To accurately simulate the damage evolution process of gouged pipelines, this study adopts an elastic–plastic constitutive model combined with the GTN damage model to describe the mechanical behavior of the material [15,16,17]. The elastic–plastic constitutive model has been obtained from the tensile tests, whereas the GTN damage model requires determination of nine parameters, namely, the model coefficients *q*_1_, *q*_2_, and *q*_3_ accounting for the interaction between neighboring voids within the material; the mean equivalent plastic strain *ε_N_*; the standard deviation of the equivalent plastic strain *S_N_*; the initial void volume fraction *f*_0_; the nucleated void volume fraction *f_N_*; the critical void volume fraction *f_c_*; and the void volume fraction at final fracture *f_F_*.

**Figure 6:**
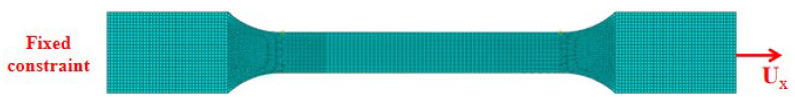
Finite element model for uniaxial tension.

To avoid excessive errors caused by mutual dependence among multiple GTN parameters, six of them are specified with reference to the studies of Acharyya et al. [18,19,20,21,22,23], namely, *q*_1_ = 1.5, *q*_2_ = 0.88, *q*_3_ = 2.25, *ε_N_* = 0.1, *S_N_* = 0.3, *f_N_ =* 0.0008. The remaining parameters *f*_0_, *f_F_* and *f_c_* are identified by finite-element inverse calibration. For this purpose, a uniaxial tensile finite element model, whose dimensions are consistent with those of the tensile specimen, is established as shown in Figure 6, and is used to back-calculate the parameters against the experimental results.

**Figure 7:**
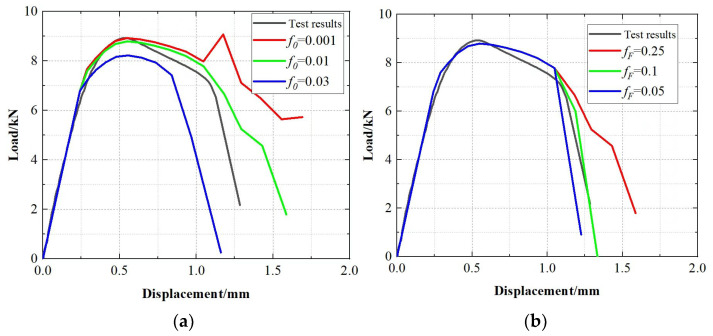
Calibration of damage parameters for X70 metamorphic layer. (**a**) *f*_0_ Effect on load-displacement curve. (**b**) *f*_F_ Effect on load-displacement curve.

**Figure 8:**
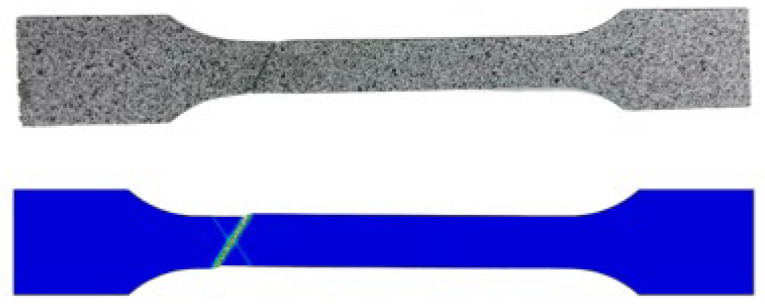
Morphology of the specimen after testing and the simulated fracture specimen.

For the damage parameters of the X70 altered layer, different parameter sets were first prescribed and evaluated against the experimental data. When *f*_0_ = 0.01, the correlation coefficient (*r*) reached a maximum value of 0.9555; therefore, *f*_0_ = 0.01 was adopted. The non-monotonic trend of r in Figure 7a arises because r measures full-curve agreement, and different parameters affect different deformation stages. Subsequently, different values of *f_F_* were tested, and when *f_F_* = 0.1, (*r*) reached its maximum value of 0.9993. Accordingly, the damage parameters were determined as *q*_1_ = 1.5, *q*_2_ = 0.88, *q*_3_ = 2.25, *f*_0_ = 0.01, *f_F_* = 0.1, *f_c_* = 0.02, *f_N_* = 0.0008, *ε_N_* = 0.3, *S_N_* = 0.1. The morphology of the specimen after the test and the images of the simulated fractured specimen are shown in Figure 8. The fracture locations and fracture surface morphologies of both are very similar, further confirming the accuracy of the obtained damage parameters. For the X70 base material, the damage parameters were calibrated using the same method, and the resulting GTN damage parameters are given in Table 2.

### 3.2. Establishment of the Pipeline Model

All finite element simulations were performed using ABAQUS/Explicit Version 2022. The geometric model of the scratched pipeline consists of two parts: the base material and the altered layer. The geometric parameters are as follows: pipe outside diameter D = 1016 mm, wall thickness t = 14.2 mm, and a selected pipe length of 5000 mm. The actual scratch dimensions were obtained by 3D scanning: scratch depth *d*_g_ = 0.82 mm and scratch width *W*_g_ = 10 mm. To reduce computational cost, the scratch length was taken as *L*_g_ = 60 mm According to the hardness test results, an altered layer with a thickness of 1.08 mm was defined at the bottom of the scratch along the thickness direction.

**Figure 9:**
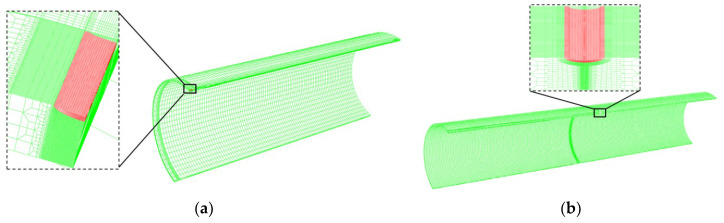
Mesh encryption at scratch defect. (**a**) axial scratch; (**b**) circumferential scratch.

In the internal pressure simulation model, the scratch is oriented in the axial direction, as shown in Figure 9a; in the combined internal-pressure-plus-tension simulation model, the scratch is oriented in the circumferential direction, as shown in Figure 9b, while the scratch dimensions remain unchanged. To reduce computational cost, the pipeline model subjected to internal pressure only is simplified to a quarter model by using symmetry with respect to the neutral plane and the tangential plane. Since an axial scratch is not sensitive to tensile loading, the tension + internal-pressure model is simplified to a half model. Symmetry boundary conditions are applied to ensure consistency of the global mechanical response.

The entire model uses three-dimensional solid reduced-integration eight-node linear brick elements (C3D8R). Mesh refinement is applied in the scratch region in both the radial and circumferential directions, with an element size of approximately 0.1 mm × 0.2 mm × 0.5 mm. In the far-field region, the mesh is gradually coarsened up to 25 mm to balance accuracy and computational cost. The final model contains more than 400,000 elements. A mesh sensitivity study was conducted by refining the element size in the gouge regio. The key global response showed only minor changes when further refining from the medium to the fine mesh (difference within 3.3%), indicating that the adopted mesh provides mesh-independent predictions at a reasonable computational cost.

During the numerical simulations, a symmetry boundary condition is imposed in the Z-direction at the pipe end near the scratch, and symmetry in the X-direction is enforced along the pipe axis. An equivalent axial tensile load is applied at the opposite end to represent the end-cap effect.

For the internal-pressure-only case, pressure is applied to the inner surface and increased gradually until a stable response is achieved. For the combined internal pressure and tensile loading case, the internal pressure is held constant while an axial displacement is applied to the cross-section at the fixed end until failure. The displacement rate is selected to ensure quasi-static loading.

To verify the accuracy of the finite element model in the non-defective region, the circumferential strain of the far-end parent material area of the pipe under internal pressure only was calculated based on thin-walled cylinder theory. The calculation yielded $\varepsilon_{\theta }$ = 0.00257, $\varepsilon_{LE}$ = ln(1 + $\varepsilon_{\theta }$) ≈ 2.57 × 10^−3^.

(1) $$\varepsilon_{\theta }=\frac{\sigma_{\theta }}{E}-\nu \frac{\sigma_{z}}{E}$$

where: $\sigma_{\theta }$ is the circumferential stress; $\sigma_{z}$ is the axial stress; *E* is the modulus of elasticity; and ν is Poisson’s ratio.

**Figure 10:**
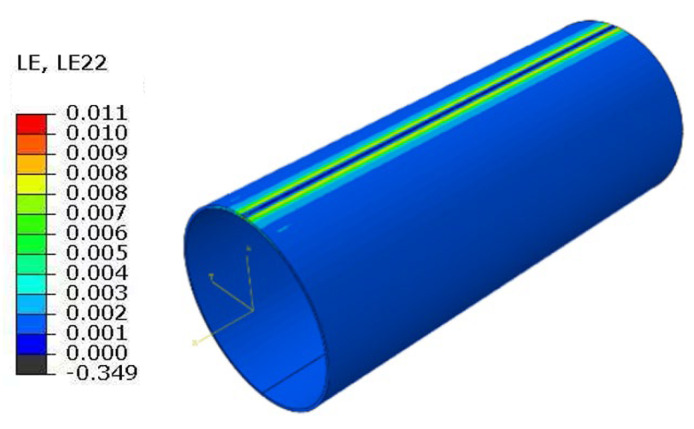
Simulation results of circumferential strain (MPa).

Representative nodes were selected on the surface of the base material, away from scratches and edge constraints (at axial distances greater than 3 L from the center of the scratch and circumferentially avoiding the meridian line where the scratch is located). The circumferential logarithmic strain LE22 was extracted in a custom cylindrical coordinate system and compared with the theoretically calculated circumferential strain. The simulation result was 2.588 × 10^−3^, as shown in Figure 10. Its strain response closely matches the theoretical value, with a deviation within 5%, indicating that the model reasonably describes the stiffness and deformation characteristics of the base material region. This provides a reliable benchmark for subsequent modeling of local degraded layers and crack initiation simulations. This validation result further supports the accuracy of the load, boundary condition, and material parameter settings in the model.

## 4. Damage Evolution Analysis of Scratched Pipelines

To investigate the effect of the altered layer on the stress distribution and failure behavior of the scratched pipeline under different loading conditions, finite element analyses were performed for two cases: (i) internal pressure only, and (ii) combined internal pressure and axial tensile loading. The internal pressure at the onset of element deletion was taken as the pipeline failure pressure.

### 4.1. Internal-Pressure-Only Condition

**Figure 11:**
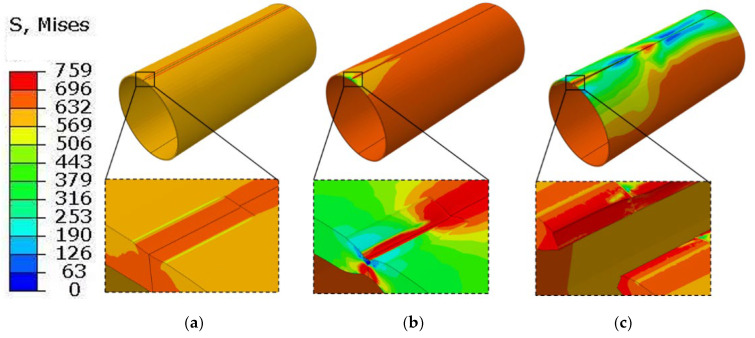
Von Mises stress distribution (MPa) of the gouged pipeline without the metamorphic layers under internal pressure. (**a**) Before crack initiation; (**b**) Crack initiation; (**c**) Radial penetration.

**Figure 12:**
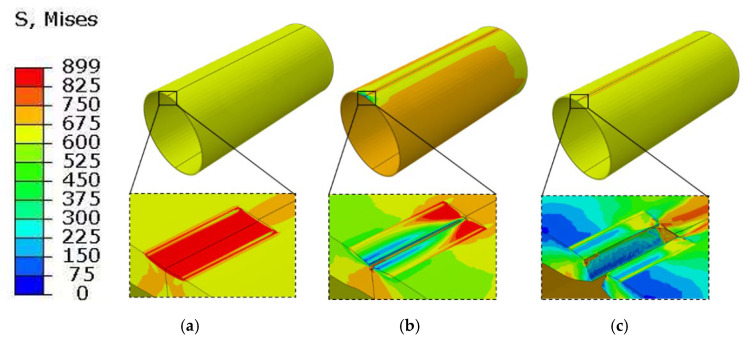
Von Mises stress distribution (MPa) of the gouged pipeline considering the metamorphic layers under internal pressure. (**a**) Before crack initiation; (**b**) Crack initiation; (**c**) Radial penetration.

Figure 11 and Figure 12 show the equivalent stress distribution of the scratched pipeline under internal pressure only, without and with consideration of the altered layer, respectively.

When the altered layer is neglected, symmetric, tongue-shaped high-stress regions form at both ends of the scratch under pure internal pressure. The stress contours remain relatively smooth, and the plastic zone gradually propagates through the wall thickness along the scratch sidewall. At the stage shown in Figure 11b, the internal pressure reaches 19 MPa, at which a crack initiates within the pipe wall at the scratch bottom. The crack then propagates through the wall thickness and, as the internal pressure increases to 19.2 MPa, begins to extend in the axial direction.

When the altered layer is included, the equivalent stress at the scratch root increases markedly under the same internal pressure, and the stress contours become substantially more concentrated. At the stage shown in Figure 12b, the internal pressure reaches 16.5 MPa, at which crack initiation occurs in the altered layer at the scratch bottom. The crack then propagates through the wall thickness and, as the internal pressure increases to 16.6 MPa, penetrates radially through the wall. A comparison of the two cases indicates that accounting for the altered layer reduces the failure pressure under internal loading by 13.2%.

### 4.2. Combined Internal Pressure and Tensile Loading

**Figure 13:**
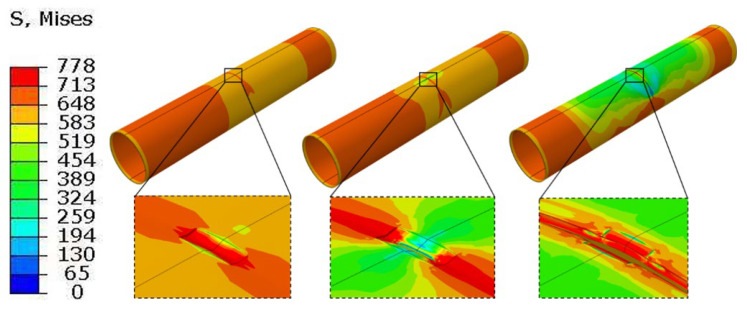
Von Mises stress distribution (MPa) of the gouged pipeline without the metamorphic layers under internal pressure.

**Figure 14:**
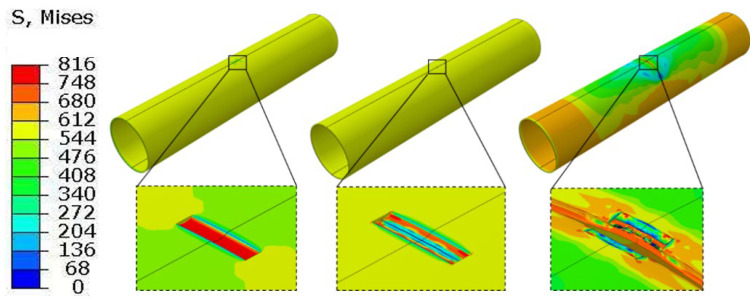
Von Mises stress distribution (MPa) of the gouged pipeline considering the metamorphic layers under internal pressure.

Figure 13 presents the stress distribution of the scratched pipeline under combined internal pressure and tensile loading when the altered layer is neglected, whereas Figure 14 shows the corresponding results when the altered layer is included. The two cases exhibit pronounced differences in the local stress–strain evolution and the associated degradation of load-bearing capacity.

When the altered layer is not considered, a relatively broad shear band develops at the scratch root. The high-stress region evolves symmetrically along the pipe axis, with a comparatively mild stress gradient, indicating effective stress redistribution. Crack initiation occurs near the mid-length of the scratch bottom and subsequently propagates stably along the symmetric shear band.

When the altered layer is considered, a much sharper stress concentration appears at the scratch root. Crack initiation localizes in the altered layer at the scratch bottom and rapidly penetrates through the wall thickness. The stage of stable crack growth is shortened, and the post-peak response becomes more abrupt (i.e., a shorter stable deformation interval before instability), indicating reduced ductility reserve, which is consistent with the reduced fracture strain of the altered layer and the GTN damage variable reaching its critical value more quickly.

**Figure 15:**
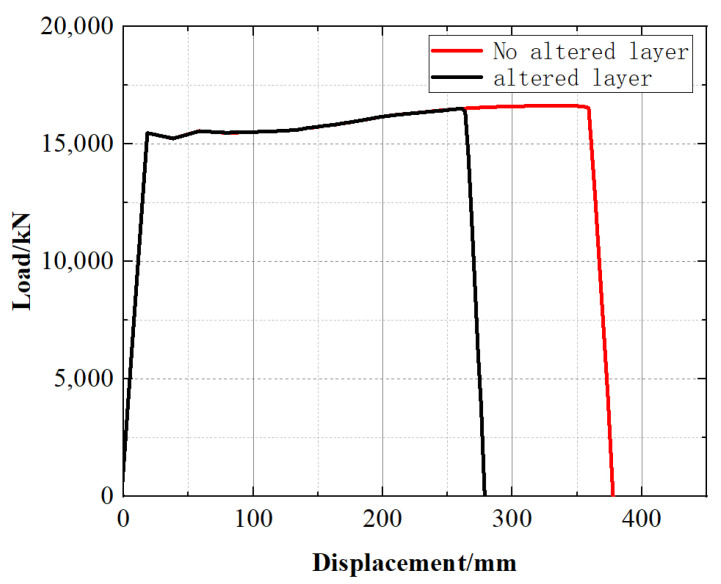
Load–displacement curves of the two models.

A comparison of the load–displacement curves for the two models (Figure 15) indicates that the peak loads are essentially identical, suggesting that the altered layer has a limited influence on the peak load-carrying capacity. In contrast, accounting for the altered layer markedly reduces the deformation capacity: instability occurs at a displacement of 263 mm, whereas the ultimate displacement reaches 358 mm when the altered layer is neglected.

Therefore, neglecting the altered layer leads to an overestimation of the instability displacement and thus a non-conservative assessment of the deformation tolerance under combined loading. The small difference in peak load explains why it is difficult to capture the effect of the material gradient using only the ultimate load as an index. It is thus recommended that, when assessing the failure risk of scratched pipelines, the altered layer at the scratch bottom be modeled as an independent material domain.

## 5. Conclusions

Based on X70 pipeline steel and focusing on the altered layer at the bottom of the scratch, this study obtains the mechanical property parameters of the altered layer through microhardness tests and tensile tests. On this basis, a finite element model of a scratched pipeline that distinguishes between base material and altered layer is established. Fracture behavior under pure internal pressure and combined internal pressure–tension loading is investigated, and the dominant influence of the altered layer on crack-initiation location and load-carrying capacity is clarified. The main conclusions are as follows:

(1) For the three characteristic cross-sections of the hardness specimens, the Vickers hardness along the thickness direction monotonically decreases from the surface toward the base material (Figure 5). The hardness in the base material region is mainly concentrated in the range of 188–230 HV, whereas the surface altered layer, strengthened by plastic deformation, shows a markedly higher hardness. Using 230 HV as a contour value allows for a stable delineation of the boundary between the altered layer and the base material, from which the average thickness of the altered layer is determined to be about 1.08 mm.

(2) Compared with the base material, the altered layer exhibits a “high-strength–low-ductility” behavior. Its peak hardness increases by 148 HV; at a thickness of 1.08 mm, the yield strength and ultimate tensile strength increase by 39% and 47%, respectively, while the uniform elongation decreases by 88.7%. The elastic modulus remains essentially unchanged, as expected for cold-work hardening, and the yield-to-tensile strength ratio increases.

(3) Under pure internal pressure loading, explicitly accounting for the altered layer shifts crack initiation to the altered layer and reduces the crack initiation pressure from 19 MPa to 16.5 MPa (−13.2%). Under combined internal pressure and tensile loading, the peak load remains almost unchanged, but the instability displacement decreases from 358 mm to 263 mm (−26.5%), indicating a pronounced reduction in ductility reserve governed by rapid damage localization in the altered layer. Therefore, neglecting the altered layer may overestimate the safety margin, particularly in terms of deformation tolerance under combined loading.

## Figures and Tables

**Table 1:** Mechanical properties of the materials.

Materials	*E* (GPa)	*R*_p0.2_ (MPa)	*R*_m_ (MPa)	*A* (%)
X70-B	202	541	610	16%
X70-M	197	752	899	1.8%

**Table 2:** Material damage parameters.

Materials	*q*_1_	*q*_2_	*q*_3_	*f*_0_	*f_F_*	*f_c_*	*f_N_*	*ε_N_*	*S_N_*
Base material	1.5	0.88	2.25	0.0001	0.25	0.02	0.0008	0.3	0.1
altered layer	1.5	0.88	2.25	0.01	0.1	0.02	0.0008	0.3	0.1

## Data Availability

The original contributions presented in the study are included in the article, further inquiries can be directed to the corresponding author.
